# The R1-weighted connectome: complementing brain networks with a myelin-sensitive measure

**DOI:** 10.1162/netn_a_00179

**Published:** 2021-04-27

**Authors:** Tommy Boshkovski, Ljupco Kocarev, Julien Cohen-Adad, Bratislav Mišić, Stéphane Lehéricy, Nikola Stikov, Matteo Mancini

**Affiliations:** NeuroPoly Lab, Polytechnique Montreal, Montreal, QC, Canada; Macedonian Academy of Sciences and Arts, Skopje, Macedonia; NeuroPoly Lab, Polytechnique Montreal, Montreal, QC, Canada; Department of Neurosciences, Faculty of Medicine, University of Montreal, Montreal, QC, Canada; Functional Neuroimaging Unit, Centre de recherche de l’institut universitaire de gériatrie de Montréal, Montreal, QC, Canada; Montreal Neurological Institute, Montreal, QC, Canada; Paris Brain Institute (ICM), Centre for NeuroImaging Research (CENIR), Inserm U 1127, CNRS UMR 7225, Sorbonne Université, F-75013, Paris, France; NeuroPoly Lab, Polytechnique Montreal, Montreal, QC, Canada; Montreal Heart Institute, Montreal, QC, Canada; NeuroPoly Lab, Polytechnique Montreal, Montreal, QC, Canada; Department of Neuroscience, Brighton and Sussex Medical School, University of Sussex, Brighton, UK; CUBRIC, Cardiff University, Cardiff, UK

**Keywords:** Connectome, Myelin, Diffusion MRI, Quantitative MRI

## Abstract

Myelin plays a crucial role in how well information travels between brain regions. Complementing the structural connectome, obtained with diffusion MRI tractography, with a myelin-sensitive measure could result in a more complete model of structural brain connectivity and give better insight into white-matter myeloarchitecture. In this work we weight the connectome by the longitudinal relaxation rate (R1), a measure sensitive to myelin, and then we assess its added value by comparing it with connectomes weighted by the number of streamlines (NOS). Our analysis reveals differences between the two connectomes both in the distribution of their weights and the modular organization. Additionally, the rank-based analysis shows that R1 can be used to separate transmodal regions (responsible for higher-order functions) from unimodal regions (responsible for low-order functions). Overall, the R1-weighted connectome provides a different perspective on structural connectivity taking into account white matter myeloarchitecture.

## Author Summary

In the present work, we show that by using a myelin-sensitive measure we can complement the diffusion MRI-based connectivity and provide a different picture of the brain organization. We show that the R1-weighted average distribution does not follow the same trend as the number of streamlines strength distribution, and the two connectomes exhibit different modular organization. We also show that unimodal cortical regions tend to be connected by more streamlines, but the connections exhibit a lower R1-weighted average, while the transmodal regions have higher R1-weighted average but fewer streamlines. This could imply that the unimodal regions require more connections with lower myelination, whereas the transmodal regions rely on connections with higher myelination.

## INTRODUCTION

The brain is a complex system that can be modelled as an intricate network of interconnected elements (Fornito et al., 2016). Using magnetic resonance imaging (MRI), connectomics aimsto characterize macroscopic connectivity by viewing the brain as a set of nodes defined by functionally or anatomically distinguishable regions of interest (ROIs) and edges that are conventionally assumed to reflect the white matter tracts connecting those nodes (Bassett & Sporns, 2017; Hagmann et al., 2007; van den Heuvel et al., 2008). Specifically, the white matter tracts can be reconstructed using diffusion MRI and tractography (Jeurissen et al., 2019; Mori & Van Zijl, 2002). To better characterize the relationship between the nodes and edges of a brain network, weights can be assigned to the connections, which are presumed to reflect relevant properties (Rubinov & Sporns, 2010).

There is an ongoing debate as to the most appropriate choice of weighting for the connectome (Yeh et al., 2020). So far, the most widely used weight is the number of streamlines (NOS), which counts the reconstructed streamlines, from diffusion tractography, between pairs of ROIs (Fornito et al., 2016). Although previous work (Sinke et al., 2018; van den Heuvel et al., 2015) showed a positive correlation between NOS and tract-tracing connectivity, suggesting that NOS could be used in principle as a proxy for microstructural fiber count, the use of NOS to weight the structural connectome is still problematic (Calamante, 2019). In particular, NOS does not measure biologically meaningful properties such as conduction velocity. Additionally, fiber tracking often lacks specificity as it can be affected by a number of factors, including the tractography algorithm used (Jones, 2010; Yeh et al., 2020) as well as image acquisition parameters (Jones et al., 2013).

Another potential candidate for weighting the connections is the fractional anisotropy (FA) that can be obtained using diffusion tensor imaging (DTI). While FA does provide more insights into the microstructural properties of white matter, it is also influenced by numerous tissue properties, including axonal diameter, fiber density, tissue geometry, as well as the degree of myelination (Jones et al., 2013). Another reason why FA might not be the best candidate for weighting the connectome is because it is derived from the same diffusion-based measures that are used to reconstruct the tractography. To gain additional insights into the myeloarchitecture it would make sense to weight the connectome by a metric that is orthogonal to diffusion. Many quantitative MRI (qMRI) measures (i.e., magnetization transfer ratio [MTR], longitudinal relaxation rate [R1], myelin water fraction [MWF]) have been used to characterize myelin. Myelin is the dielectric material that wraps around the axons to enable fast conduction in the brain. The use of such metrics is particularly well suited for studies that examine activity-dependent myelination (Sampaio-Baptista & Johansen-Berg, 2017) and pathology related to myelin-specific changes in brain connectivity.

Several studies (Caeyenberghs et al., 2016; Kamagata et al., 2019; Mancini et al., 2018; van den Heuvel et al., 2010) used such myelin-sensitive MR measures in brain network models. Specifically, in the work of Caeyenberghs et al. (2016), multiple quantitative myelin-sensitive MRI metrics were used as weights, including the R1, which has been shown to be effective for myelin imaging (Stüber et al., 2014). Caeyenberghs et al. analyzed the white matter plasticity using connectomics to determine which measures best correlate with white matter plasticity during a working memory task. To weight the connectome they used diffusion-derived measures (FA, AD, 1/MD, 1/RD, TRF [total restricted fraction], TVF [tissue volume fraction], MWF), and relaxometry measures (R1 and R2). They reported that the increased global efficiency in the network during working memory tasks was best captured by the R1-weighted connectome. The influence of myelin on R1 values can be traced to its molecular composition. Voxels containing more myelinated axons have an increased proportion of macromolecules, which increases the longitudinal relaxation rate (Yeatman et al., 2014). Although R1 is affected by iron, calcium content, and axon size (Harkins et al., 2016) and count (Schmierer et al., 2008), a recent meta-analysis showed that R1 is comparable to other MRI techniques for quantifying myelin content (Mancini et al., 2020).

In this article, we introduce a myelin-sensitive measure (R1) to the structural connectome. We do this by weighting the connections in the structural connectome using the median R1 value along a bundle of streamlines connecting pairs of brain regions. We then compared the R1-weighted connectome with the conventional NOS-weighted connectome in terms of multiple network attributes, including strength distribution and modular structure. The differences between the R1- and NOS-weighted connectomes in terms of their overall network organization have the potential to provide a complementary perspective on white matter myeloarchitecture, as R1 is more directly sensitive to myelin compared to NOS.

## MATERIALS AND METHODS

### Data Acquisition

Thirty-five healthy volunteers (HC) (12 female/23 male, mean age ± *SD*: 61.2 ± 9.16 years) participated in the present study. Subjects were scanned at the Paris Brain Institute (ICM – Institut du Cerveau), Paris, France. All subjects signed informed consent forms. The study was approved by the local ethics committee (Ethics Committee: Comité de Protection des Personnes [CPP] Ile de France VI - RCB: 2014-A00725-42). Scans were performed on a 3T SIEMENS Prisma Scanner. The protocol included (i) 3-shell DWI sequence (TR = 10,400 ms, TE = 59 ms, voxel size = 1.7 × 1.7 × 1.7 mm^3^, number of gradient directions per shell = 64, 32, and 8 at, respectively, b = 2,500, 700, and 300 s/mm^2^) and (ii) magnetization-prepared 2 rapid acquisition gradient echoes (MP2RAGE) sequence for R1 mapping (TR = 5,000 ms, TE = 2.98 ms, flip angles = 4° and 5°, TI = 700/2,700 ms, FOV = 256 × 232 mm, voxel size = 1 mm^3^).

### Reconstruction of Quantitative R1 Maps

The MP2RAGE sequence (Marques et al., 2010) produces two T1-weighted images with different flip angles and different inversion times (INV1 and INV2). These images are then combined to produce a more uniform T1w image (UNI). The UNI image was used to estimate the longitudinal relaxation times (T1 maps) using qMRLab (Karakuzu et al., 2020). The longitudinal relaxation rate (R1) was then calculated from the T1 maps as:

$$R_{1}=\frac{1}{T_{1}}$$

The quantitative maps were reconstructed using the qMRLab module MP2RAGE (Karakuzu et al., 2020).

### Anatomical and Diffusion Data Preprocessing

As a first step in the anatomical preprocessing pipeline, background noise removal (O’Brien et al., 2014) was applied to the UNI images by using a combination of the two inversion time images with a denoising regularization factor of 70. The denoised UNI images were then processed using FreeSurfer 6.0 (Fischl, 2012) to segment the different tissues and parcellate the brain using the Desikan–Killiany Atlas (Desikan et al., 2006). To reduce the bias from the different parcel sizes, we subdivided them into finer regions of approximately equal size using the Lausanne 2008 parcellation (scale 125) (Cammoun et al., 2012; Hagmann et al., 2008), which resulted in 234 brain parcels. Furthermore, because this article focuses on the connectivity between cortical regions, we discarded all the subcortical regions from the analysis, which resulted in 219 brain regions.

The preprocessed anatomical images, T1w image, and parcellation; in addition the reconstructed quantitative maps for each subject were transferred to the subject’s diffusion space by coregistering them to the mean b0 image using FSL FLIRT (Jenkinson et al., 2002, 2012) rigid body registration. Each registration was visually inspected to check the alignment (see Figure S1 in the Supporting Information). Besides the registration, all preprocessed images were visually inspected for errors.

The preprocessing of the diffusion images was done using MRtrix3 (Tournier et al., 2019). First, we applied a noise removal technique (Veraart et al., 2016a, 2016b) followed by a Gibbs ringing artifacts removal method (Kellner et al., 2016) and a B1 field inhomogeneity correction. Then, the images were preprocessed for motion and inhomogeneity distortion correction using FSL’s *eddy* (Andersson & Sotiropoulos, 2016) and *topup* tools (Andersson et al., 2003), respectively. Furthermore, to increase the anatomical contrast and improve the tractography and registration, the preprocessed images were upsampled to a 1-mm isotropic resolution. Multitissue constrained spherical deconvolution (Jeurissen et al., 2014), followed by the anatomically constrained tractography method (Smith et al., 2012), were used to reconstruct the tractogram. We applied the SD_STREAM deterministic tracking algorithm (Tournier et al., 2012) that used 1 million seeds dynamically placed using the SIFT model (Smith et al., 2015). The tractography procedure was set to stop either when (i) it produces 200,000 streamlines and/or (ii) the maximum number of seeds (1,000,000) is reached. During tracking the maximum turning angle was set to 60°. Streamlines with length shorter than 20 mm or longer than 250 mm were discarded from the tractogram. Additional constraints were provided by the anatomically constrained tractography (ACT) framework (Smith et al., 2012).

### Structural Connectome Reconstruction

Structural connectivity was represented using a weighted graph, where each node corresponded to one of the 219 cortical ROIs, and each edge reflected the presence of reconstructed streamlines between each pair of ROIs. Two metrics were used as weights of the connections: (i) the NOS reconstructed between two regions and (ii) the median R1 values along the bundle of reconstructed streamlines between two regions. The same steps were followed to reconstruct the FA-weighted connectome (see Supporting Information). We decided to use the median value of the metric (R1 or FA) along the tract for two main reasons: (i) the median is less sensitive to outliers and (ii) it does not assume a normal distribution of the values along the bundle.

To mitigate the problem with spurious connections reconstructed by the tractography algorithm, we considered two nodes as connected only if there are at least two streamlines connecting the specific pair of ROIs. Also, a more conservative threshold (at least five connections) was applied to test the robustness of the results.

A group consensus approach for both NOS- and R1-weighted connectomes was adopted to reduce individual variability in the reconstructed networks. The group consensus networks for both connectomes were constructed by taking into account only the connections that are present in at least 50% of the subjects (de Reus & van den Heuvel, 2013). The weight of a connection in the group consensus network corresponded to the median of the connection’s weights across subjects. We then assessed the relationship between the connection’s weights of the R1-weighted connectome and the NOS-weighted connectome using linear regression, as well as between the R1-weighted connectome and the FA-weighted connectome.

### NOS Strength and R1-Weighted Average

We chose strength as a measure of centrality because of its straightforward interpretation. For the NOS-weighted connectome, the strength was calculated as:

$$S_{i}^{NOS}=\overset{N}{\underset{j}{\sum }}w_{ij}$$

where *i* is a given node, and *w*_*ij*_ is the NOS connectivity between the nodes *i* and *j*.

For the R1-weighted connectome, we looked at the R1-weighted average, as it is not influenced by the number of connections (Kamagata et al., 2019). The R1-weighted average was calculated as:

$$S_{i}^{R1}=\frac{\sum_{j}^{N}w_{ij}v_{ij}}{\sum_{j}^{N}w_{ij}}$$

where *i* is a given node, *w*_*ij*_ is the number of streamlines and *v*_*ij*_ is the median R1 sampled along the bundle of those streamlines connecting the nodes *i* and *j*.

We then looked at the distribution of the centrality measures for each weight. The nodes were first sorted according to their NOS strength. Then, we defined the hubs as regions that have NOS strength of at least 2 standard deviations above the mean NOS strength van den Heuvel & Sporns, 2013. A more conservative hub definition, at least 3 standard deviations above the mean NOS strength, was also used. Then, we highlighted the hub regions, defined in the NOS-weighted connectome and in the R1-weighted connectome.

### Modular Structure

To probe the modular structure of the NOS- and R1-weighted connectomes, we used a modularity maximization method (Blondel et al., 2008; Rubinov & Sporns, 2011; Sporns & Betzel, 2016). This is a common method that is used to divide a network into modules/communities with highly interconnected regions within, and less connected regions between the submodules. To achieve this, the method aims to maximize a quality function given by the following equation:

$$Q(\gamma )=\overset{N}{\underset{ij}{\sum }}\left[A_{ij}-\gamma P_{ij}\right]\delta \left(c_{i},c_{j}\right)$$

where *A*_*ij*_ is the empirical connectivity matrix, and *P*_*ij*_ represents the estimated connectivity matrix given a specific null model. The module assignment of node *i* is described by the variable *c*_*i*_, whereby *δ*(*c*_*i*_, *c*_*j*_) is the Kronecker function which is equal to 1 when *c*_*i*_ = *c*_*j*_ and 0 otherwise.

The modularity maximization also depends on a resolution parameter (*γ*), which makes it sensitive to different scales. If *γ* < 1, then the network is partitioned into larger modules, while for *γ* > 1 the method tends to find smaller modules.

To determine at which resolution the modular structure is best described, that is, when it maximizes the quality functions, for each connectome we iterated over *γ* values ranging from 0.5 to 3 with steps of 0.1. At each step, we ran the Louvain algorithm 1,000 times (Blondel et al., 2008). Then, the resolution parameter (*γ*) with highest Q was selected on the basis of the highest Rand index (Traud et al., 2011) similarity and created a consensus modularity using the netneurotools package (Netneurolab, 2020).

### Rank-Based Analysis

To further explore the modular structure and assess the difference between weights, a rank-based analysis (Vázquez-Rodríguez et al., 2019) was performed: the nodes were first sorted by their strength (for the NOS-weighted connectome) and by their weighted average (for the R1-weighted connectome) defining their nodal rank (1 meaning highest and 219 meaning lowest). Then, nodal ranks in the NOS-connectome were subtracted from the corresponding nodal ranks in the R1-weighted connectome. To normalize the difference, a **z** score normalization was applied. The nodes were then grouped according to the von Economo cytoarchitectonic parcellation (Scholtens et al., 2018) and Yeo’s functional parcellation (Yeo et al., 2011). Finally, the median *z* score for each cytoarchitectonic and functional class was computed across the respective nodes.

## RESULTS

**Figure 1.**
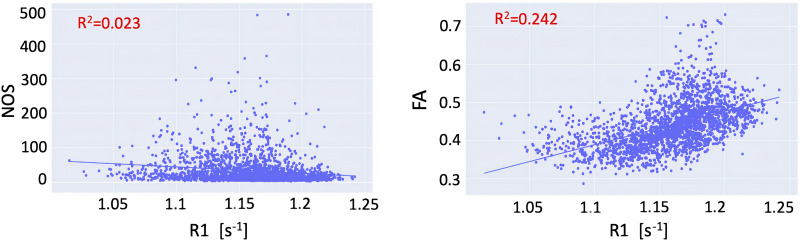
Relationship between the connection weights in the R1-weighted and FA-weighted connectome (left) and R1-weighted and NOS-weighted (right).

To assess the shared variance between the different connectomes, we first compared the connection weights of the R1-weighted connectome with the weights obtained from the NOS-and FA-weighted connectomes. We found that the R1 and NOS weights exhibited an *R*^2^ of 0.023 (*p* < 0.01), while the R1 and the FA weights exhibited *R*^2^ of 0.24 (*p* < 0.01) (Figure 1). Given that R1 measures different microstructural properties compared to NOS and FA, the shared variance between the connections weighted with these measures is limited.

**Figure 2.**
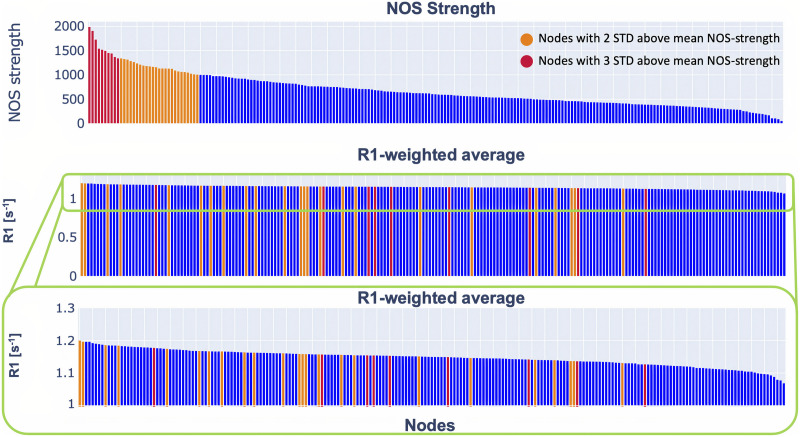
Distribution of the NOS strength and R1-weighted average. The plot in the middle shows the distribution of the R1-weighted average on a scale from 0 to 1.2. To make it easier to see the trend, we showed the same data on a scale from 1 to 1.2 (bottom plot). In orange are highlighted the nodes that are 2 standard deviations above the mean NOS strength, while in red are highlighted the nodes that are 3 standard deviations above the NOS strength. The details about the nodes are provided in the Supporting Information.

Next, we looked at the strength distribution and weighted average for the NOS- and R1-weighted connectomes. The strength distribution of the NOS-weighted connectome is heavy tailed (Figure 2). Among the nodes with the highest strength were the superior frontal gyrus, lateral occipital, pre-, and postcentral gyrus. (Table S1 in the Supporting Information).

The R1-weighted average distribution did not follow the same trend as the NOS strength distribution (Figure 2). This result indicates that a high number of streamlines is not associated with higher R1 values. Also, the hubs defined with the more conservative threshold (at least 3 standard deviations above the mean NOS strength) did not exhibit a high R1-weighted average (Figure 2).

**Figure 3.**
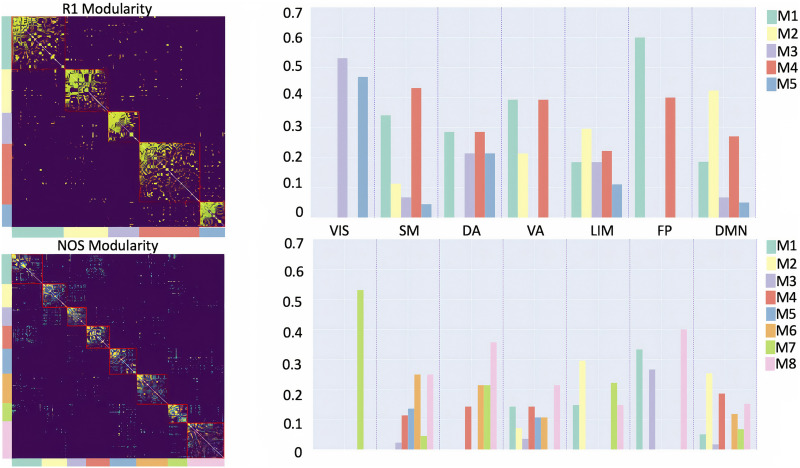
Community structure of the R1- and NOS-weighted connectomes. The bar plots represent the distributions of functional classes, given by Yeo et al. (2011), within the modules (denoted as M#) for the R1- and NOS-weighted connectomes, respectively. Yeo’s functional classes include the following: SM (somatomotor), VIS (visual), VA (ventral attention), FP (fronto-parietal), LIM (limbic), DA (dorsal attention), and DMN (default mode network).

As for the community structure (Figure 3), the selected resolution parameter was 0.8 for the R1-weighted, while for the NOS-weighted connectome it was 2.6. The consensus modularity for the R1-weighted connectome yielded 5 modules with average modularity score *Q*(*γ*) = 0.569, whereas the NOS-weighted connectome yielded 11 modules with an average modularity score of *Q*(*γ*) = 0.44. We further explored the organization of the modules by looking at the distributions of the functional classes of the nodes provided by Yeo et al. (2011). Both the NOS and R1 modules were found to include multiple functional classes.

**Figure 4.**
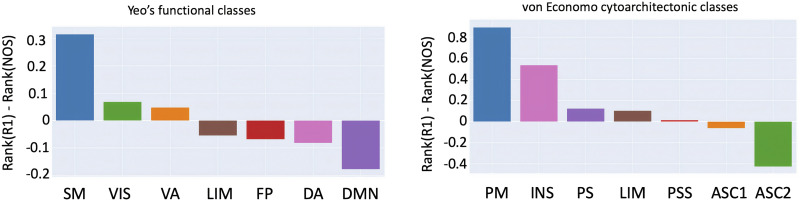
Rank-based comparison across functional and cytoarchitectonic classes. The rank for each node was calculated by its strength (for NOS)/weighted average (for R1) and then grouped using a cytoarchitectonic parcellation and a functional one. Yeo’s functional classes include the following: SM (somatomotor), VIS (visual), VA (ventral attention), FP (fronto-parietal), LIM (limbic), DA (dorsal attention), and DMN (default mode network). Von Economo cytoarchitectonic classes include the following: PM (primary motor), INS (insular), LIM (limbic), PS (primary sensory), PSS (primary secondary sensory), ASC1 (association cortex), and ASC2 (association cortex 2).

The rank-based analysis (Figure 4) shows where the functional and cytoarchitectonic classes are over- and underrepresented in terms of R1-weighted average and NOS strength. For Yeo’s functional atlas, the R1 is overrepresented (compared to NOS) in the higher-order subnetworks (transmodal) and underrepresented for function-specific subnetworks (unimodal).However, this is not the case for the cytoarchitectonic subnetworks derived using the von Economo parcellation, that is, the transmodal/unimodal distinction was less obvious, as R1 was also underrepresented for the insular and the limbic subnetworks, which are transmodal.

We repeated the same analysis on the connectomes constructed with a stricter threshold, that is, two regions are connected if there are at least five streamlines reconstructed between them (see Supporting Information). The results showed that centrality measures’ distributions and rank-based analysis are consistent between the two thresholds. However, regarding the modularity, R1-based connectome yielded a different number of modules, although the community structure was still different from NOS. Furthermore, the same analysis was done on the connectome constructed using probabilistic tractography and we have obtained similar results (see Supporting Information).

## DISCUSSION

In this study, we showed that by using a myelin-sensitive measure we can complement the diffusion MRI-based connectivity and provide a different picture of the brain organization. To better characterize the myelin-weighted connectome, we decided to compare it with a connectome weighted by a diffusion-based metric. While there were several candidates for comparison, such as apparent axon density (Raffelt et al., 2012) and SIFT2 (Smith et al., 2015), we settled on NOS as it is the most commonly used approach.

First, we focused on the strength distribution and compared it to the R1-weighted average. From Figure 2 one can appreciate that they do not follow the same trend. The R1-weighted average reflected a more uniform distribution. We also found that the hub regions, defined in the NOS connectome, do not necessarily have a high R1-weighted average. Similar results have been previously reported in Mancini et al. (2018) for a g-ratio-weighted connectome.

Second, we observed differences in the modular structure between the NOS- and R1-weighted connectomes. The number of modules was influenced by the resolution parameter, and a different number of modules was expected as the most optimal parameters were different for the two connectomes. However, what we wanted to highlight in this study was the different modular structure for the two weights, and to do this we partitioned the network in the most appropriate way for each weight. We also explored the distribution of the functional classes within the modules and found that there was limited agreement between the functional classes and the estimated modules, that is, the modules included multiple functional classes. This result is in agreement with results previously reported in the literature: it has been observed that structural and functional perspectives highlight different interregional relationships (Betzel et al., 2013; Goñi et al., 2014; Honey et al., 2010; Suárez et al., 2020).

Regarding the rank-based analysis, we found that there was a good division of the unimodal versus transmodal functional classes. This pattern seems to follow the functional gradient observed in previous studies (Margulies et al., 2016; Vázquez-Rodríguez et al., 2019). An interesting result was that the unimodal regions exhibited more connections but in proportion a lower R1-weighted average, while the transmodal regions exhibited a higher R1-weighted average but less connections. A recent study has shown an opposite trend in cortical gray matter (Glasser & van Essen, 2011), but our study focuses on white matter connectivity and uses a different imaging modality (R1 versus T1w/T2w).

Our results showed that differences exist between the connectome weighted with NOS and the one weighted with R1 in terms of the distribution of their weights, as well as in the modular organization. Interestingly, the rank-based analysis showed an agreement in subdivision of the regions in unimodal and transmodal functional subnetworks. Future studies could focus on the relationship between white matter myeloarchitecture and function.

The use of qMRI metrics to weight the connectome could have important implications for many applications. qMRI offers several techniques that are sensitive to myelin (Laule et al., 2007; Petiet et al., 2019), such as magnetization transfer, myelin water imaging, or relaxometry (for extensive reviews see Heath et al., 2018, and Piredda et al., 2020). Additionally, these techniques could be used to estimate the conduction velocity and conduction delays, and to incorporate these metrics as weights in the connectome. This would potentially result in a more complete model of the structural connectome and may provide a more comprehensive understanding of how the structure shapes the function. In this direction, Berman and colleagues calculated the conduction delay among the fibers in the corpus callosum using MRI-derived g-ratio (Berman et al., 2019). However, to calculate the conduction velocities and delays, in addition to the information about myelin, one would also need information about the axonal diameter and potentially information about other microstructural properties not accessible from MRI (Drakesmith et al., 2019). The work of Drakesmith et al. (2019) studied the feasibility of estimating conduction velocity in vivo using MRI microstructural measures. They performed simulations and reported that most of the variance in the estimation of the conduction velocity is explained by the axonal diameter and the g-ratio. However, axonal diameter can be accurately measured only with high gradients (300 mT/m) (Veraart et al., 2020) and is therefore not a measure that one can have on a clinical scanner yet. Additionally, even at such high gradients, the MRI-derived axonal measure is not sensitive to small axons (1 μm or lower) (Jones et al., 2018), so there are still challenges that need to be tackled in order to compute a robust estimate of the conduction velocity or delay.

There are a few methodological aspects of this work that are worth mentioning. The first is the choice of quantitative MRI metrics to weight the connectome (Collin et al., 2014, 2016; Larivière et al., 2019; Messaritaki et al., 2019; Verstraete et al., 2011). As mentioned before, the structural connectome is often weighted using diffusion-derived metrics such as NOS and FA. For NOS, this stems from the assumption that streamline count is a proxy of microstructural fiber count, that is, the greater the number of streamlines, the higher the connectivity between regions. This has been shown to be questionable, however, as results are influenced by the tractography algorithms and the choice of tracking parameters. Here we decided to use R1 to weight the connectome, as it has been repeatedly shown to correlate highly with myelin content (Lee et al., 2012; Lutti et al., 2014). Also, the MP2RAGE sequence, which was used to acquire the R1 maps, is a stock, relatively short protocol with open-source processing, which makes it suitable for a wide clinical application. There are several studies that demonstrated the usefulness of complementing the tractography with longitudinal relaxation time. For instance, De Santis et al. (2014) showed that to compare two groups, that is, to detect differences between groups, the longitudinal relaxation time (T1), which is just an inverse of R1, requires a smaller sample size compared to the diffusion derived metrics. Another study (De Santis et al., 2016) demonstrated that it is possible to measure tract-specific T1 relaxation, potentially leading to fiber-specific myelin metrics and more thorough network models.

Another aspect is that here we weighted the connectome using the median rather than the standard approach of taking the mean along the bundle of reconstructed streamlines. This is due to the fact that the median is more robust against outliers and does not rely on the normality assumption for the R1 distribution along a fiber bundle. Relying on one measure per bundle instead of averaging a measure across streamlines also avoids biasing the results towards NOS.

Furthermore, we should also mention the choice of network measures that were investigated. The more canonical graph measures such as clustering coefficient and path length were not calculated. The rationale behind our choice is the consequence of the complex role of myelin in white matter pathways: myelinated axons show faster conduction than unmyelinated axons, but when comparing myelinated axons with different amount of myelin, the overall effect on conduction speed, as already mentioned, depends on several factors (e.g., axon diameter, g-ratio, internodal) (Drakesmith et al., 2019). Therefore, the use of myelin measures in network models requires more careful interpretation. As the weight in network models usually reflects the intensity or capacity of a connection (Barrat et al., 2004), most analyses rely on the same assumption. To take into account the myelin-specific role in conduction phenomena and to avoid the strict intensity-based interpretation of the network weight, we decided to focus on the use of R1-weighted average as a centrality measure (as previously done in Kamagata et al., 2019) and on the modular structure.

The choice of tractography algorithm is also an important methodological aspect that has to be considered when reconstructing the connectome. Two main classes of tractography algorithms, deterministic and probabilistic, can be employed to reconstruct the connectome. There is an ongoing debate on the advantages and disadvantages of these two classes of algorithms for mapping the connectome (Sarwar et al., 2019). Both classes of algorithms are valid choices for performing fiber tracking but they also have some disadvantages: in recent studies (Maier-Hein et al., 2017; Sotiropoulos & Zalesky, 2019), it has been shown that the deterministic tractography algorithms reconstruct fewer true positive streamlines compared to probabilistic tractography. On the other hand, probabilistic tractography reconstructs more false positive streamlines as opposed to deterministic tractography, which further biases the reconstructed connectome by introducing spurious connections. In this study, we used both deterministic and probabilistic algorithms to double check the robustness of our results. The limitation of introducing spurious connections is particularly evident for modularity: the different results obtained for R1 using two different thresholds may imply that including spurious streamlines deeply affects the R1 weight distribution and therefore the estimated modular structure. To tackle these thresholding issues, new algorithms have recently been proposed (Schiavi et al., 2020; Smith et al., 2015) that aim to reduce the number of false positive streamlines by using microstructural and anatomical priors. Future studies need to clarify how such methods could be applied to combine tractography with complementary measures.

Finally, one limitation of this study is the relatively small sample size. Unfortunately, we are not aware of any publicly available dataset that includes quantitative MRI metrics (besides diffusion derived ones) that are sensitive to myelin.

In conclusion, the R1-weighted connectome complements the structural connectome derived from dMRI and could provide new biomarkers for many pathologies that affect the brain. Further validation of this approach is required, for example, by studying demyelinating diseases.

## ACKNOWLEDGMENTS

We thank the ICEBERG study group and particularly Marie Vidailhet, MD (Pitié-Salpêtrière Hospital, Paris, Principal investigator), Jean-Christophe Corvol, MD, PhD (Paris Brain Institute, Paris, clinical and genetic data), Isabelle Arnulf, MD, PhD (Pitié-Salpêtrière Hospital, Paris, clinical and sleep data), Rahul Gaurav, MS, (Pitié-Salpêtrière Hospital, Paris, data analysis), Nadya Pyatigorskaya, MD, PhD, (Pitié-Salpêtrière Hospital, Paris, data analysis); for their help in collecting data.

## SUPPORTING INFORMATION

Supporting information for this article is available at https://doi.org/10.1162/netn_a_00179. In the Supporting Information we provided additional results using also the FA to weight the connectome. We also reported the outcomes obtained using a more stringent threshold in the connectivity matrices as well as outcomes obtained using probabilistic tractography to reconstruct the connectomes. The code and data to reproduce the results are available on GitHub (https://github.com/TommyBoshkovski/The_R1-weighted_connectome, Boshkovski, 2020).

## AUTHOR CONTRIBUTIONS

Tommy Boshkovski: Conceptualization; Formal analysis; Methodology; Visualization; Writing – original draft; Writing – review & editing. Ljupco Kocarev: Conceptualization; Methodology; Writing – review & editing. Julien Cohen-Adad: Conceptualization; Methodology; Writing – review & editing. Bratislav Misic: Conceptualization; Methodology; Writing – review & editing. Stéphane Lehéricy: Conceptualization; Data curation; Methodology; Writing – review & editing. Nikola Stikov: Conceptualization; Methodology; Supervision; Writing – review & editing. Matteo Mancini: Conceptualization; Methodology; Supervision; Writing – review & editing.

## FUNDING INFORMATION

Stéphane Lehéricy, Investissements d’Avenir, Award ID: ANR-10-IAIHU-06. Stéphane Lehéricy, Investissements d’Avenir, Award ID: ANR-11-INBS-0006. Stéphane Lehéricy, EDF Foundation. Nikola Stikov, Fondation Institut de Cardiologie de Montréal (http://dx.doi.org/10.13039/501100012651). Nikola Stikov, Canadian Open Neuroscience Platform (Brain Canada PSG). Nikola Stikov, Réseau en Bio-Imagerie du Quebec (http://dx.doi.org/10.13039/100010571), Award ID: 8436-0501. Nikola Stikov, Natural Sciences and Engineering Research Council of Canada (http://dx.doi.org/10.13039/501100000038), Award ID: 2016-06774. Nikola Stikov, Fonds de Recherche du Québec - Santé (http://dx.doi.org/10.13039/501100000156), Award ID: FRSQ 36759 and FRSQ 35250. Matteo Mancini, Wellcome Trust (http://dx.doi.org/10.13039/100004440), Award ID: 213722/Z/18/Z. Stéphane Lehéricy, Fondation Thérèse and René Planiol.

## TECHNICAL TERMS

Myeloarchitecture: Spatial organization of myelinated axons in the cerebral cortex.
qMRI: Quantitative MRI, referring to the use of MRI to measure tissue physical and chemical properties.
Myelin: Lipidic structure that wraps around axons and allows faster electrical conduction.
Flip angle (in magnetic resonance): Angle at which an RF pulse tips the longitudinal magnetization into the transverse plane.
Inversion time (in magnetic resonance): Time between an 180 (inversion) pulse and 90 (excitation) pulse.
Longitudinal relaxation time (in magnetic resonance): Time constant of the exponential decay of the net magnetization longitudinal component after an excitation pulse.
Constrained spherical deconvolution: Mathematical approach to deconvolve the diffusion MRI signal into fiber orientationspecific spherical harmonics.
Unimodal regions: Brain regions responsible for specific, concrete functions.
G-ratio: Ratio of the inner and the outer diameters of a myelinated axon.
Transmodal regions: Brain regions involved in multiple- or higher-level functions.

## External Supplement

https://github.com/TommyBoshkovski/The_R1-weighted_connectome
