# Supplementary material for: The R1-weighted connectome: complementing brain networks with a myelin-sensitive measure
Source: Netw Neurosci. 2021 Apr 27;5(2):358–72. doi: 10.1162/netn_a_00179 (PMC8233108; doi:10.1162/netn_a_00179)
Supplement: Supplementary file 1 [file netn-05-358-s001.pdf]

Boshkovski, T., Kocarev, L., Cohen-Adad, J., Mišić, B., Lehéricy, S., Stikov, N., & Mancini, M. (2020). Supporting information for "The R1-weighted connectome: complementing brain networks with a myelin-sensitive measure." *Network Neuroscience*. Advance publication. https://doi.org/10.1162/netn_a_00179

The R1-weighted connectome: complementing brain networks with a myelin-sensitive measure

Tommy Boshkovski[1], Ljupco Kocarev[2], Julien Cohen-Adad[1,3,4], Bratislav Mišić[5], Stéphane Lehéricy[6], Nikola Stikov[1,7,a], Matteo Mancini[1,8,9,a,*]

[1] NeuroPoly Lab, Polytechnique Montreal, Montreal, Canada;

[2] Macedonian Academy of Sciences and Arts, Skopje, Macedonia;

[3] Department of Neurosciences, Faculty of Medicine, University of Montreal, Montreal, QC, Canada;

[4] Functional Neuroimaging Unit, Centre de recherche de l'institut universitaire de gériatrie de Montréal, Montreal, QC, Canada;

[5] Montreal Neurological Institute, Montreal, QC, Canada;

[6] Paris Brain Institute (ICM), Centre for NeuroImaging Research (CENIR), Inserm U 1127, CNRS UMR 7225, Sorbonne Université, F-75013, Paris, France;

[7] Montreal Heart Institute, Montreal, QC, Canada;

[8] Department of Neuroscience, Brighton and Sussex Medical School, University of Sussex, Brighton, United Kingdom;

[9] CUBRIC, Cardiff University, Cardiff, United Kingdom.

[a] These authors contributed equally to this work.

[*] Corresponding author

Supplementary Materials

S1. Quality assurance of the data

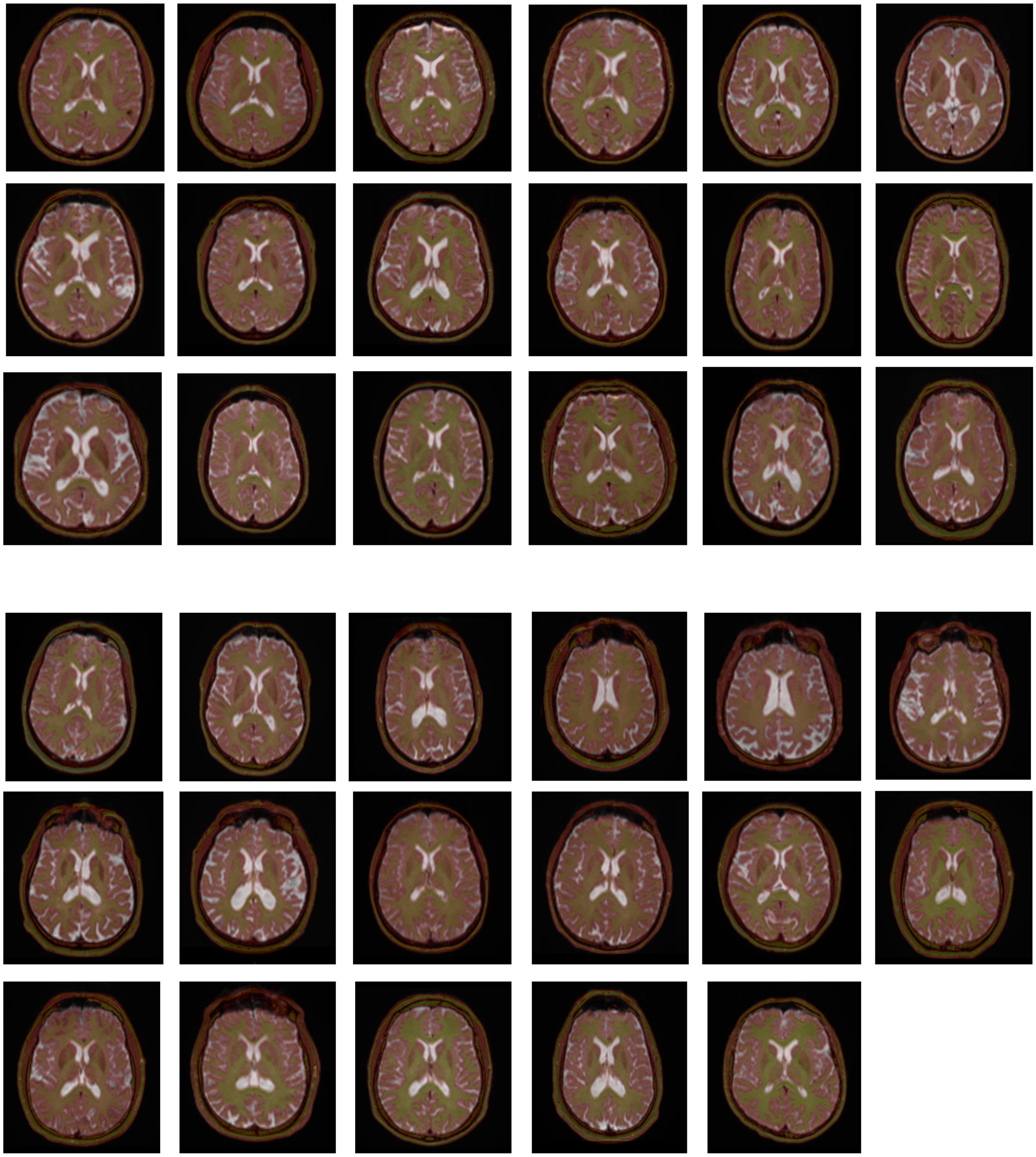

Figure S1. Overlay of the registered T1-weighted volume (in red) over the mean b0 volume (in gray scale) for all subjects (representative slices).

S2. Additional Analyses

Comparison with FA-weighted networks

As an additional comparison, we built a FA-weighted connectome. The procedure for obtaining the FA-weighted network is the same as for the R1-weighted connectome: we assigned to each connection the median FA value along the bundle of streamlines connecting pairs of regions.

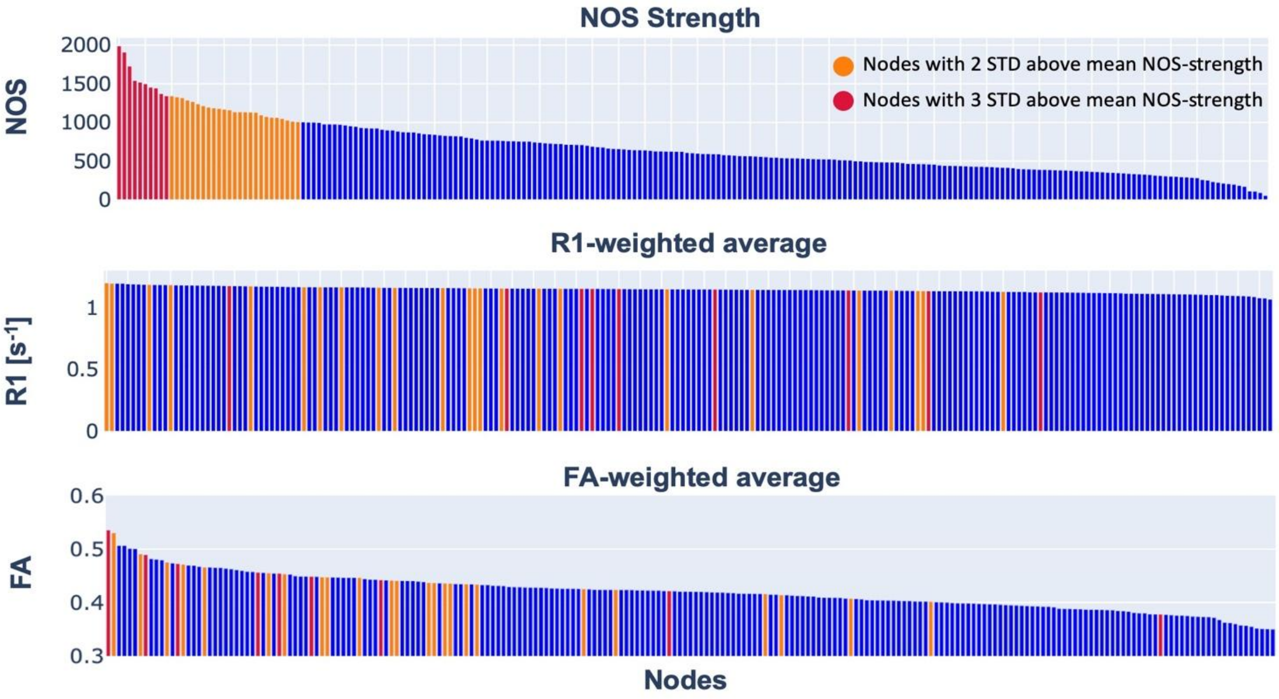

Figure S2. Strength and weighted average distribution of the group NOS-, FA-, and R1-weighted connectome. In orange are highlighted the nodes that are two standard deviations above the mean NOS-strength, while in red are highlighted the nodes that are three standard deviations above the NOS-strength.

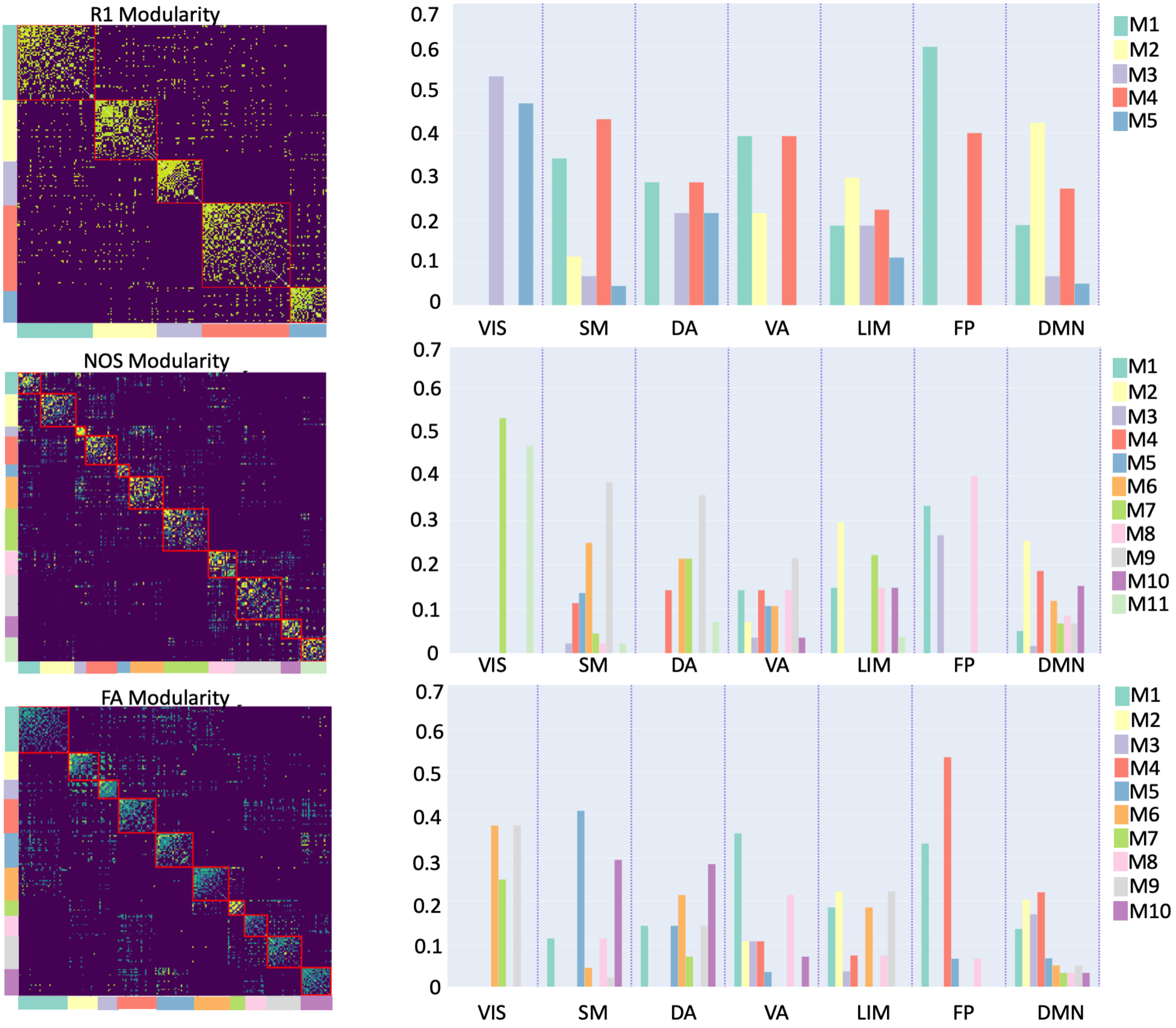

Figure S3. Community structure of the R1-, FA-, and NOS-weighted connectomes. The bar plots represent the distributions of functional classes, given by Yeo et al., within the modules (denoted as M#) for the R1-, NOS-, and FA-weighted connectomes, respectively. Yeo's functional classes: SM (Somatomotor), VIS (Visual), VA (Ventral Attention), FP (Frontoparietal), LIM (Limbic), DA (Dorsal Attention), DMN (Default Mode Network)

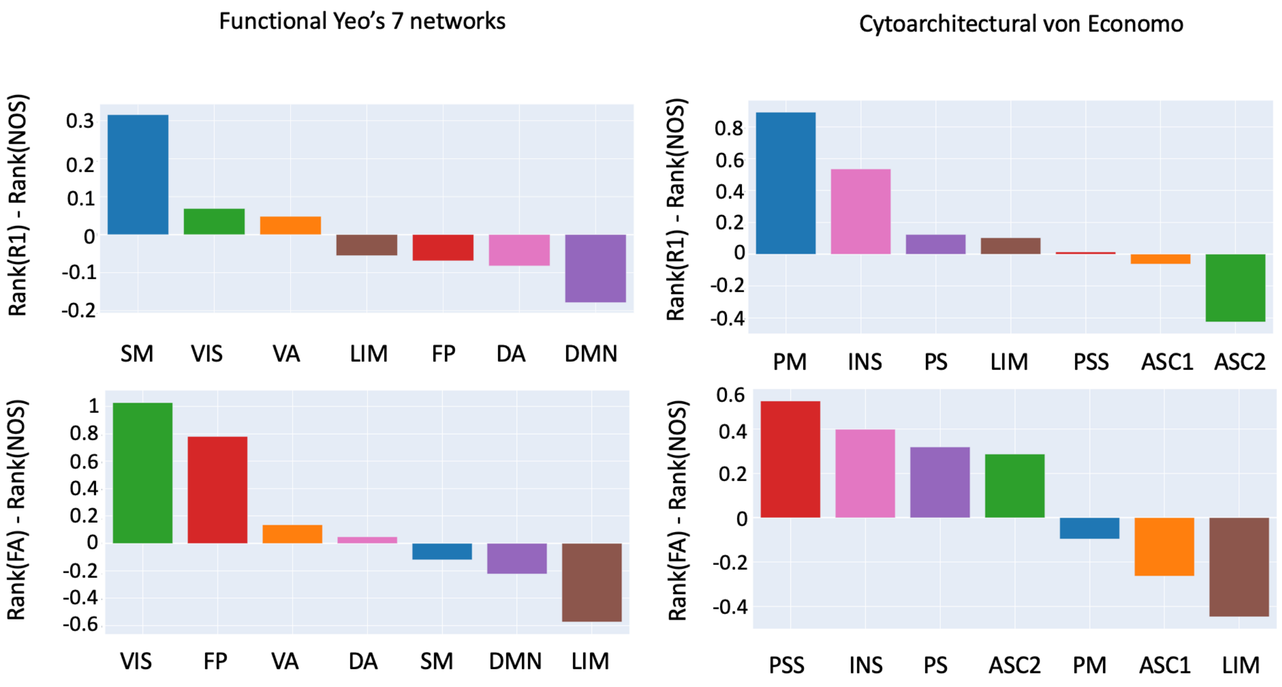

Figure S4. Gradient of the nodes' rank. The rank for each node was calculated by its strength (for NOS) and weighted average (for R1 and FA). Then the nodes were grouped using a cytoarchitectonic parcellation and functional one. Yeo's functional classes: SM (Somatomotor), VIS (Visual), VA (Ventral Attention), FP (Frontoparietal), LIM (Limbic), DA (Dorsal Attention), DMN (Default Mode Network). Von Economo cytoarchitectonic classes: PM (primary motor), INS (insular), LIM (Limbic), PS (primary sensory), PSS (primary secondary sensory), ASC1 (association cortex), ASC2 (association cortex 2)

Robustness of the analysis

To check the robustness of the analyses, we built a connectome that was constructed using a more conservative threshold. Basically, we considered two nodes to be connected if and only if they are at least 5 streamlines reconstructed between them.
The order of the figures mimics the same order as the figures in the manuscript.

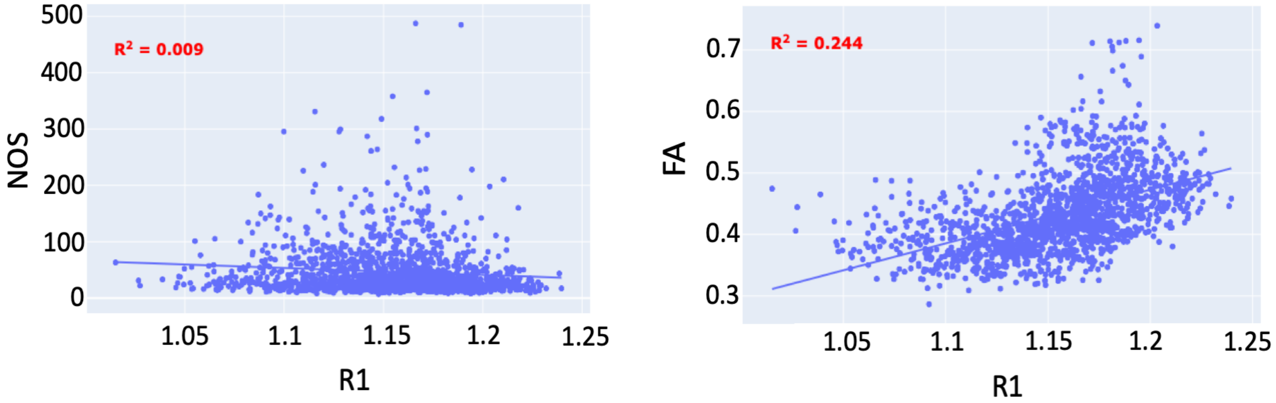

Figure S5. Relationship between the connection weights in the R1-weighted and FA-weighted connectome (left) and R1-weighted and NOS-weighted (right)

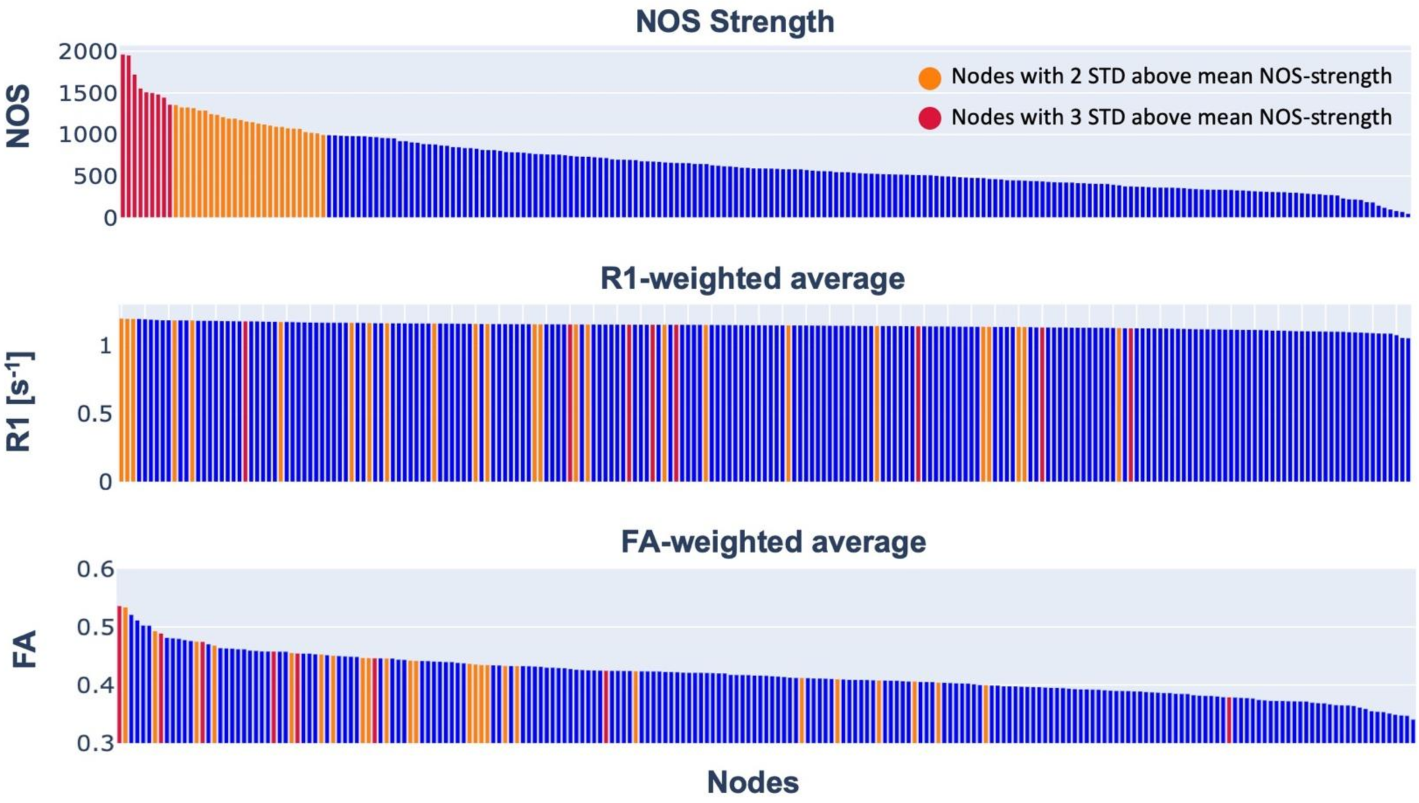

Figure S6. Strength and weighted average distribution of the group NOS-, FA-, and R1-weighted connectome. In orange are highlighted the nodes that are two standard deviations above the mean NOS-strength, while in red are highlighted the nodes that are three standard deviations above the NOS-strength.

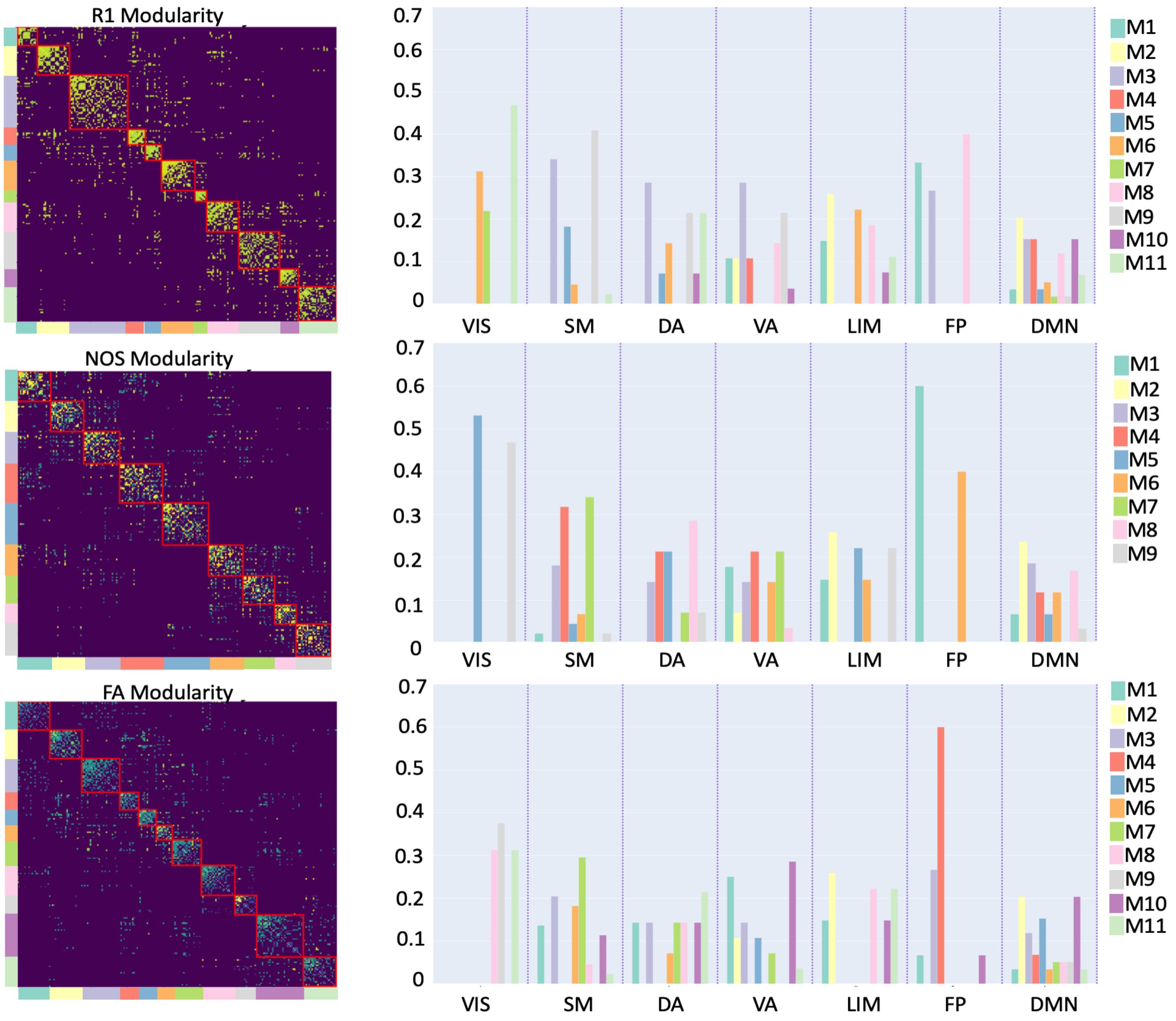

Figure S7. Community structure of the R1-, FA-, and NOS-weighted connectomes. The selected resolution parameter was 2.8 for the R1-weighted connectome, 2.6 for the FA-weighted connectome, and 2 for the NOS-weighted connectome. The bar plots represent the distributions of functional classes, given by Yeo et al., within the modules (denoted as M#) for the R1-, NOS-, and FA-weighted connectomes, respectively. Yeo's functional classes: SM (Somatomotor), VIS (Visual), VA (Ventral Attention), FP (Frontoparietal), LIM (Limbic), DA (Dorsal Attention), DMN (Default Mode Network)

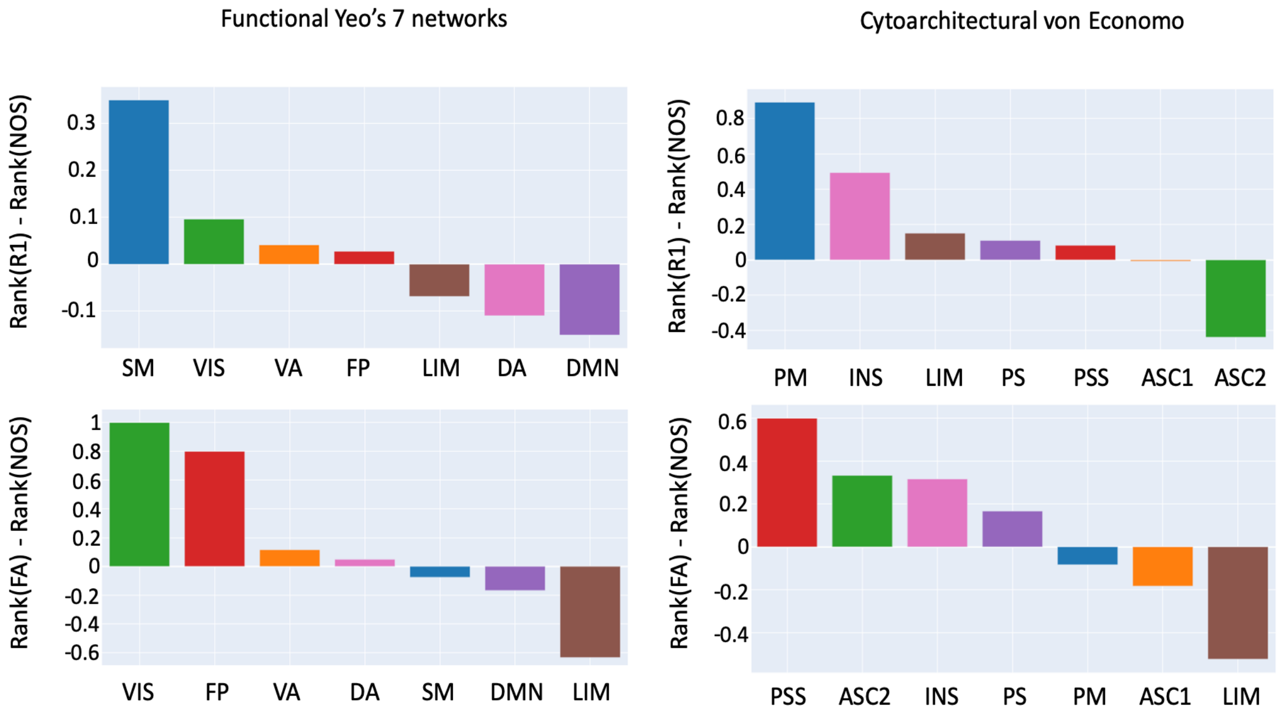

Figure S8. Gradient of the nodes' rank. The rank for each node was calculated by its strength (for NOS) and weighted average (for R1 and FA). Then the nodes were grouped using a cytoarchitectonic parcellation and functional one. Yeo's functional classes: SM (Somatomotor), VIS (Visual), VA (Ventral Attention), FP (Frontoparietal), LIM (Limbic), DA (Dorsal Attention), DMN (Default Mode Network). Von Economo cytoarchitectonic classes: PM (primary motor), INS (insular), LIM (Limbic), PS (primary sensory), PSS (primary secondary sensory), ASC1 (association cortex), ASC2 (association cortex 2)

Probabilistic tractography

To further check the robustness of the analyses, a Second-order Integration over Fiber Orientation Distributions (iFOD2) probabilistic tractography algorithm was employed to reconstruct the connectomes.

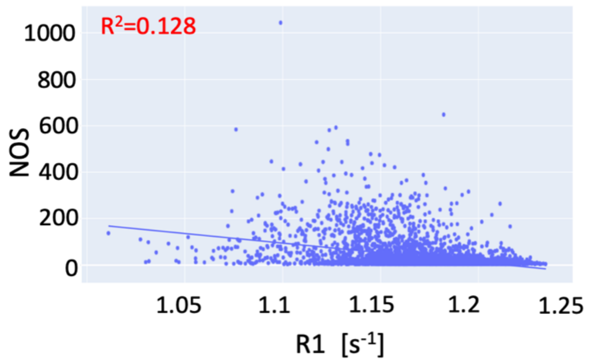

Figure S9. Relationship between the connection weights in the R1-weighted and FA-weighted connectome (left) and R1-weighted and NOS-weighted (right) reconstructed with probabilistic tractography.

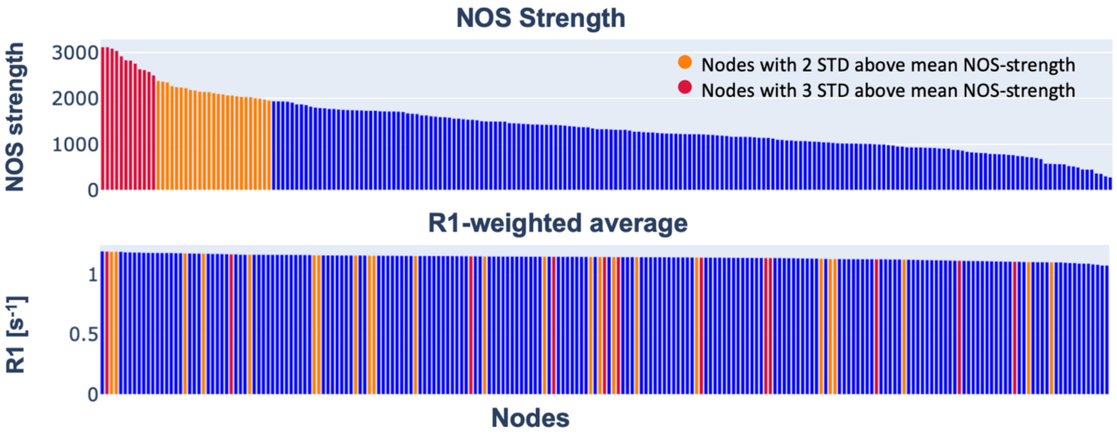

Figure S10. Strength and weighted average distribution of the group NOS- and R1-weighted connectome reconstructed with probabilistic tractography. In orange are highlighted the nodes that are two standard deviations above the mean NOS-strength, while in red are highlighted the nodes that are three standard deviations above the NOS-strength.

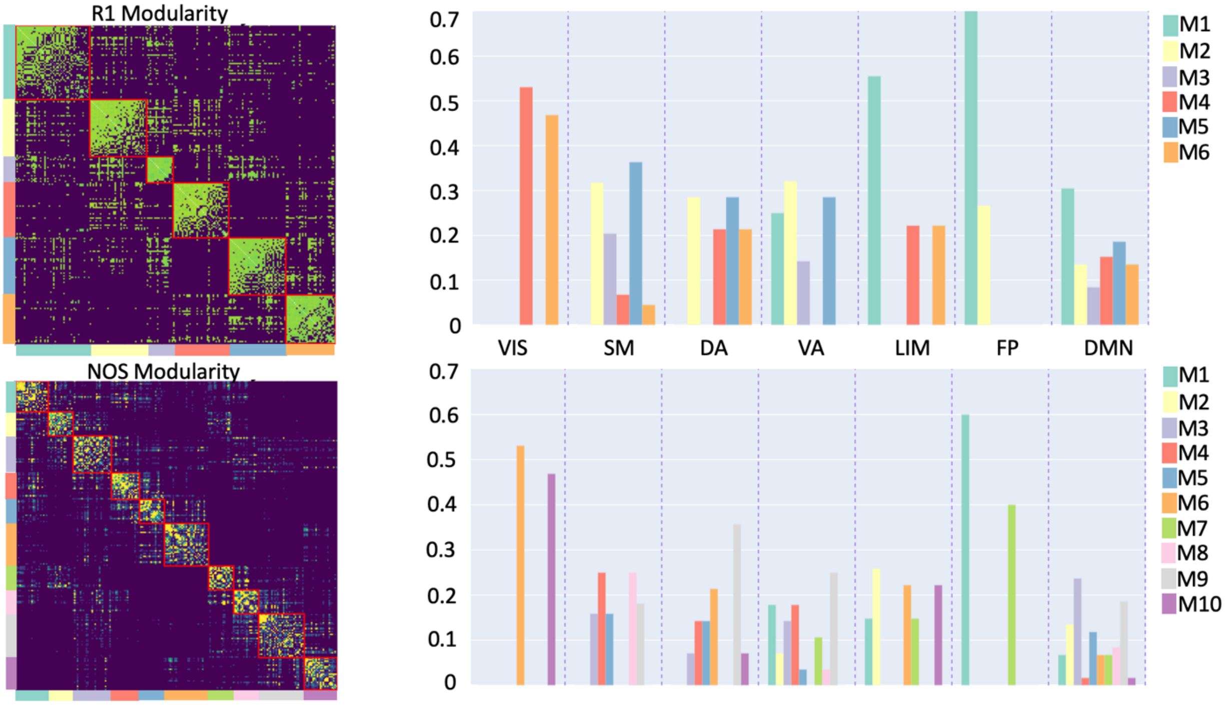

Figure S11. Community structure of the R1-, FA-, and NOS-weighted connectomes reconstructed with probabilistic tractography. The selected resolution parameter was 1.6 for the R1-weighted connectome, and 1.8 for the NOS-weighted connectome. The bar plots represent the distributions of functional classes, given by Yeo et al., within the modules (denoted as M#) for the R1- and NOS-weighted connectomes, respectively. Yeo's functional classes: SM (Somatomotor), VIS (Visual), VA (Ventral Attention), FP (Frontoparietal), LIM (Limbic), DA (Dorsal Attention), DMN (Default Mode Network)

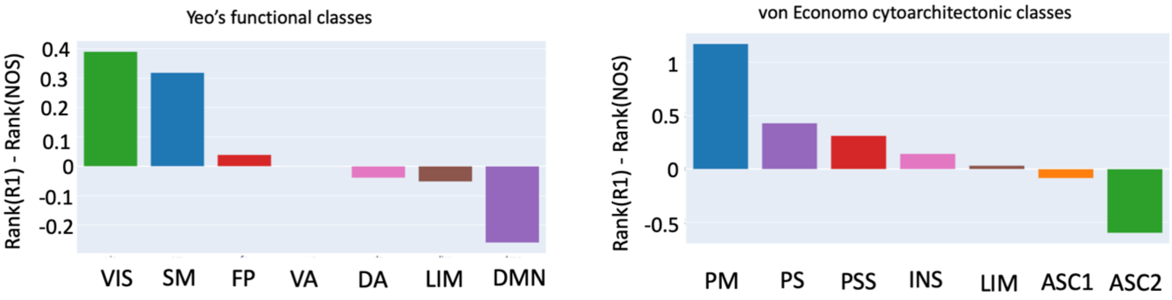

Figure S12. Gradient of the nodes' rank. The rank for each node was calculated by its strength (for NOS) and weighted average (for R1). Then the nodes were grouped using a cytoarchitectonic parcellation and functional one. Yeo's functional classes: SM (Somatomotor), VIS (Visual), VA (Ventral Attention), FP (Frontoparietal), LIM (Limbic), DA (Dorsal Attention), DMN (Default Mode Network). Von Economo cytoarchitectonic classes: PM (primary motor), INS (insular), LIM (Limbic), PS (primary sensory), PSS (primary secondary sensory), ASC1 (association cortex), ASC2 (association cortex 2)

S3. Strength and weighted-average distributions

Table S1. Strength and weighted average of the NOS-, R1-, and FA-weighted connectome.

	NOS Strength	R1-weighted average	FA-weighted average
caudalmiddlefrontal_3_R	1966	1.15455223	0.45481415
superiorfrontal_8_L	1955.5	1.15489756	0.5364118
superiorfrontal_5_R	1725.5	1.15657259	0.48931146
lateraloccipital_4_R	1558	1.17902009	0.37937757
caudalmiddlefrontal_3_L	1510.5	1.1425173	0.45794132
precentral_6_R	1502	1.12808163	0.47504907
lateraloccipital_1_L	1484.5	1.15372432	0.4250611
postcentral_5_R	1447	1.13491831	0.44638249
caudalmiddlefrontal_1_L	1360.5	1.15249975	0.44708179
superiorfrontal_4_R	1357.5	1.14975925	0.44246636
lateraloccipital_2_R	1330	1.18572172	0.40010313
superiorparietal_7_R	1329.5	1.18490026	0.46868563
superiorfrontal_6_L	1321	1.13927463	0.43705957
superiorfrontal_3_R	1292.5	1.19858478	0.45097488
inferiorparietal_6_R	1292.5	1.15621096	0.42422677
parsopercularis_2_L	1249.5	1.15720478	0.44614924
paracentral_2_L	1241	1.13771093	0.44208708
rostralmiddlefrontal_2_R	1214.5	1.16733895	0.43347641
superiorfrontal_6_R	1193.5	1.15941077	0.53443044

precentral_4_R	1193.5	1.13806138	0.44677296
superiorparietal_1_R	1178.5	1.16853382	0.43314363
superiorfrontal_7_L	1159	1.15768024	0.49319574
rostralmiddlefrontal_1_R	1151	1.14500352	0.41263682
cuneus_1_L	1135.5	1.13002028	0.41040971
precentral_7_L	1124	1.15641855	0.43493235
inferiorparietal_4_L	1111	1.16574541	0.43595524
lateraloccipital_4_L	1095	1.16091219	0.40645647
middletemporal_1_L	1094	1.17478661	0.45568292
middletemporal_1_R	1077.5	1.19980463	0.43495825
superiorfrontal_3_L	1075	1.16314845	0.45295976
superiorfrontal_9_L	1071.5	1.13961489	0.47527627
rostralmiddlefrontal_3_R	1032	1.15389897	0.40840144
lateraloccipital_5_R	1023.5	1.19707436	0.40468686
inferiorparietal_1_L	1017	1.1632152	0.42050431
precentral_5_R	998	1.12466084	0.44015975
superiorparietal_2_L	995.5	1.1501535	0.42405723
parstriangularis_1_L	994	1.15047862	0.41179135
inferiorparietal_5_L	988	1.16492729	0.43319758
isthmuscingulate_1_L	985.5	1.15111918	0.46342115
superiorparietal_7_L	983.5	1.15835931	0.46232216
precentral_3_L	982.5	1.10873801	0.45172339
superiorfrontal_7_R	981	1.14491754	0.50266414
supramarginal_5_L	975.5	1.15672519	0.42411832

parsopercularis_2_R	971	1.15793737	0.44405594
postcentral_3_R	961.5	1.14229538	0.41660898
rostralmiddlefrontal_2_L	960	1.15095653	0.42127552
superiorfrontal_2_R	957	1.15379185	0.45018773
paracentral_2_R	923.5	1.13875271	0.4811363
superiorfrontal_1_R	923	1.15586281	0.44184927
rostralmiddlefrontal_4 _L	908.5	1.16348021	0.39311237
superiorfrontal_5_L	905	1.15523623	0.45805608
superiorparietal_5_L	889.5	1.16252581	0.43204665
superiorfrontal_8_R	887.5	1.14141001	0.44379638
caudalmiddlefrontal_1_R	883	1.14609962	0.44162341
pericalcarine_1_L	871	1.11404583	0.39693474
superiorparietal_4_R	866.5	1.18072143	0.42485591
lateraloccipital_1_R	852	1.15844078	0.42304646
lateraloccipital_5_L	849	1.13991544	0.37970863
superiorfrontal_1_L	839.5	1.17374917	0.45768595
supramarginal_4_L	839	1.16224559	0.4302635
inferiorparietal_2_L	832	1.16368641	0.42498126
inferiortemporal_4_L	818.5	1.15592484	0.4324534
precentral_4_L	815.5	1.10742643	0.43777023
cuneus_2_R	815	1.15082102	0.44632162
rostralmiddlefrontal_5 _L	807.5	1.17197196	0.39257267
rostralmiddlefrontal_1_L	792.5	1.16495753	0.42939455
insula_3_L	790	1.10283158	0.4169084

isthmuscingulate_1_R	788.5	1.16147066	0.45335847
inferiortemporal_4_R	784.5	1.19627428	0.421529
parstriangularis_2_R	776	1.15943345	0.41145212
precentral_8_L	768	1.13441633	0.42563866
superiortemporal_5_R	767	1.14868755	0.44976691
superiorparietal_4_L	763	1.16261628	0.44885281
inferiorparietal_2_R	761	1.18345119	0.41581574
insula_2_R	760.5	1.11639998	0.42300816
precentral_2_R	754.5	1.16312108	0.44005198
rostralanteriorcingulate_1_L	745.5	1.1462204	0.42977588
supramarginal_2_R	739.5	1.19077522	0.4249869
precuneus_5_R	737	1.16825677	0.46423564
inferiorparietal_1_R	736.5	1.18330042	0.43022905
inferiorparietal_3_R	730.5	1.19412587	0.43864252
supramarginal_3_R	724	1.17614717	0.40946224
posteriorcingulate_1_L	721.5	1.14034405	0.44099281
medialorbitofrontal_2_L	704.5	1.12784405	0.41264181
precentral_5_L	699.5	1.18694673	0.45829026
inferiorparietal_4_R	699.5	1.13859579	0.42150329
supramarginal_1_R	697	1.16800185	0.42261709
parsopercularis_1_L	695	1.15456888	0.4239968
precentral_2_L	680.5	1.11527065	0.45777004
rostralanteriorcingulate_1_R	680	1.13511194	0.4101604
superiorparietal_3_L	678	1.14502659	0.40302768

paracentral_1_L	674	1.13882637	0.48018474
insula_3_R	668.5	1.08889984	0.39622527
inferiorparietal_5_R	663	1.17867113	0.39738851
supramarginal_2_L	660.5	1.15205014	0.40175146
rostralmiddlefrontal_4 _R	660	1.15286239	0.3900672
superiorfrontal_2_L	658.5	1.1514495	0.44912618
postcentral_5_L	649.5	1.1855542	0.38959689
fusiform_3_R	649.5	1.1262997	0.38848968
caudalmiddlefrontal_2_L	647.5	1.15461277	0.42479
lingual_3_R	633.5	1.15286475	0.39020298
middletemporal_3_L	627	1.15709588	0.42177641
paracentral_3_R	619.5	1.14703501	0.41685414
superiortemporal_3_L	618.5	1.14588912	0.39757772
superiorparietal_5_R	612.5	1.18296281	0.42387247
precentral_3_R	604.5	1.15056573	0.42631798
lateralorbitofrontal_1_L	602.5	1.1397014	0.36462349
rostralmiddlefrontal_3_L	595	1.16592398	0.40353479
parsopercularis_1_R	593.5	1.15170139	0.42685304
lingual_1_L	593.5	1.12772303	0.39827912
lateralorbitofrontal_2_R	592	1.11875655	0.38789434
medialorbitofrontal_1_R	590	1.14103453	0.42591573
rostralmiddlefrontal_5 _R	586	1.15616708	0.39314514
precuneus_2_L	585	1.15801093	0.44607199
lateraloccipital_3_R	585	1.15698507	0.35949723

postcentral_4_L	584	1.10408324	0.41106983
postcentral_3_L	575.5	1.11841811	0.40030039
precentral_1_L	569.5	1.12317379	0.48183509
posteriorcingulate_2_R	564	1.15623144	0.42528901
superiorfrontal_4_L	563	1.15482501	0.45464763
caudalanteriorcingulate_1_R	562	1.13303653	0.42056307
fusiform_4_L	551.5	1.13483781	0.43284576
caudalanteriorcingulate_1_L	551.5	1.12405917	0.42149176
fusiform_2_L	551	1.14184332	0.38525191
pericalcarine_1_R	544	1.13457765	0.38293242
fusiform_2_R	537.5	1.17439895	0.37263486
precentral_1_R	534	1.14323869	0.42110269
supramarginal_4_R	531	1.14736172	0.36603971
lateraloccipital_3_L	529	1.14020491	0.3774293
superiorparietal_3_R	526	1.18642675	0.43409549
precuneus_4_R	524	1.17751237	0.4590447
lingual_1_R	521.5	1.15579823	0.37019401
postcentral_7_L	520.5	1.12540779	0.37327419
fusiform_1_R	520	1.1691619	0.34769522
postcentral_1_L	517.5	1.141686	0.47087571
fusiform_1_L	515.5	1.13122178	0.34963292
superiorparietal_6_R	513.5	1.1832551	0.40556838
parsorbitalis_1_R	512.5	1.14404038	0.38672418

precuneus_3_L	506.5	1.16370884	0.46374493
superiortemporal_5_L	501.5	1.11135013	0.4596908
fusiform_4_R	500	1.16894616	0.40645676
middletemporal_2_L	495.5	1.17035314	0.45461105
middletemporal_4_R	489	1.16885257	0.41759972
rostralmiddlefrontal_6_L	485	1.17903179	0.40397749
cuneus_1_R	481.5	1.15410335	0.38543692
inferiorparietal_3_L	481	1.14867184	0.40587203
lateralorbitofrontal_2_L	478	1.12837932	0.38516694
medialorbitofrontal_1_L	468	1.15228688	0.42814361
superiorparietal_1_L	466.5	1.16181001	0.51192899
inferiortemporal_3_L	463.5	1.12694042	0.41518689
paracentral_1_R	452	1.14627292	0.46229125
rostralmiddlefrontal_6_R	451	1.15557603	0.38230397
superiortemporal_1_L	450.5	1.15113884	0.40836503
precuneus_1_L	448.5	1.16877606	0.50295654
precuneus_5_L	442.5	1.13045906	0.39570302
postcentral_2_R	442	1.14496349	0.39419567
lateralorbitofrontal_4_L	439	1.16620957	0.41169347
superiortemporal_3_R	432	1.15778882	0.38670591
superiorparietal_6_L	429.5	1.13243551	0.38101755
lateraloccipital_2_L	426	1.11668654	0.37404023
posteriorcingulate_2_L	425	1.12638331	0.39814242
posteriorcingulate_1_R	424.5	1.13274037	0.41780136

lingual_2_R	418	1.14083757	0.34839059
fusiform_3_L	417	1.13187888	0.37801114
inferiortemporal_1_R	412.5	1.15713411	0.40578383
supramarginal_3_L	410.5	1.15168448	0.4131758
superiortemporal_4_L	410.5	1.1323127	0.4079409
superiortemporal_2_R	407.5	1.17260683	0.38742364
superiorparietal_2_R	398	1.18109749	0.52135756
insula_1_L	393	1.10346004	0.3695119
postcentral_1_R	379	1.14983243	0.43441182
middletemporal_4_L	379	1.13664358	0.37230518
bankssts_1_R	375	1.17994353	0.392723
precentral_6_L	373.5	1.10567908	0.37237818
lingual_2_L	369.5	1.12052779	0.36558653
caudalmiddlefrontal_2_R	364	1.14904611	0.4179389
insula_1_R	363.5	1.13455426	0.37337997
precuneus_3_R	363	1.16865013	0.40939818
precuneus_4_L	362.5	1.15330462	0.40953028
superiortemporal_1_R	360.5	1.16366432	0.38950814
lateralorbitofrontal_3_L	357.5	1.14780076	0.3412609
lingual_4_L	351	1.11990501	0.35407521
precuneus_2_R	346	1.16974181	0.4330711
supramarginal_1_L	342.5	1.12020763	0.354774
insula_2_L	341	1.10332633	0.41775601
frontalpole_1_R	340.5	1.17891993	0.47641935

parsorbitalis_1_L	340	1.15781349	0.37857484
bankssts_1_L	338	1.14517744	0.39097622
middletemporal_2_R	336.5	1.18898979	0.41242934
middletemporal_3_R	332.5	1.17653929	0.40450314
inferiortemporal_2_L	332	1.11673579	0.40807257
medialorbitofrontal_2_R	325	1.12000391	0.40876957
postcentral_6_L	319	1.12089407	0.38173278
inferiortemporal_1_L	316.5	1.1110917	0.3982293
lingual_3_L	314.5	1.10730392	0.37253977
superiortemporal_2_L	311.5	1.14632003	0.39161942
precuneus_1_R	309.5	1.14464919	0.39955046
pericalcarine_2_R	309	1.10683551	0.36736579
bankssts_2_L	303.5	1.15361921	0.41426406
inferiortemporal_2_R	303	1.14625646	0.39568039
postcentral_2_L	297.5	1.09886111	0.44066292
lateralorbitofrontal_1_R	291	1.08972515	0.39367714
lateralorbitofrontal_3_R	285	1.11050945	0.37323283
parahippocampal_1_R	284.5	1.13200874	0.37900699
transversetemporal_1_L	276	1.1177173	0.3515679
parahippocampal_1_L	274.5	1.09368955	0.3970375
frontalpole_1_L	269.5	1.18547491	0.47789238
inferiortemporal_3_R	235	1.15869384	0.39527496
lateralorbitofrontal_4_R	224.5	1.12652473	0.39936488
postcentral_4_R	221.5	1.14408016	0.39030834

transversetemporal_1_R	216	1.13834961	0.3555768
parstriangularis_1_R	190	1.09673742	0.38169027
insula_4_L	185.5	1.07750943	0.3689944
medialorbitofrontal_3_R	147.5	1.08856092	0.3654355
temporalpole_1_L	122	1.09801082	0.40922319
superiortemporal_4_R	101	1.055135	0.374883
entorhinal_1_L	82.5	1.05744297	0.40704466
entorhinal_1_R	71.5	1.09199238	0.40333935
temporalpole_1_R	49	1.10473714	0.361872

S4. Details on the ICEBERG study group

Steering committee: Marie Vidailhet, MD, PhD, (Pitié-Salpêtrière Hospital, Paris, principal investigator of ICEBERG), Jean-Christophe Corvol, MD, PhD (Pitié-Salpêtrière Hospital, Paris, scientific lead), Isabelle Arnulf, MD, PhD (Pitié-Salpêtrière Hospital, Paris, member of the steering committee), Stéphane Lehericy, MD, PhD (Pitié-Salpêtrière Hospital, Paris, member of the steering committee);

Clinical data : Marie Vidailhet, MD, PhD, (Pitié-Salpêtrière Hospital, Paris, coordination), Graziella Mangone, MD, PhD (Pitié-Salpêtrière Hospital, Paris, co-coordination), Jean-Christophe Corvol, MD, PhD (Pitié-Salpêtrière Hospital, Paris), Isabelle Arnulf, MD, PhD (Pitié-Salpêtrière Hospital, Paris), Jonas Ihle, MD (Pitié-Salpêtrière Hospital, Paris), Caroline Weill, MD, (Pitié-Salpêtrière Hospital, Paris), David Grabli, MD, PhD (Pitié-Salpêtrière Hospital, Paris); Florence Cormier-Dequaire, MD (Pitié-Salpêtrière Hospital, Paris); Louise Laure Mariani, MD, PhD (Pitié-Salpêtrière Hospital, Paris), Bertrand Degos, MD, PhD (Avicenne Hospital, Bobigny);

Neuropsychological data : Richard Levy, MD (Pitié-Salpêtrière Hospital, Paris, coordination), Fanny Pineau, MS (Pitié-Salpêtrière Hospital, Paris, neuropsychologist), Julie Socha, MS (Pitié-Salpêtrière Hospital, Paris, neuropsychologist), Eve Benchetrit,

MS (La Timone Hospital, Marseille, neuropsychologist), Virginie Czernecki, MS (Pitié-Salpêtrière Hospital, Paris, neuropsychologist);

Eye movement : Sophie Rivaud-Pechoux, PhD (ICM, Paris, coordination); Elodie Hainque, MD, PhD (Pitié-Salpêtrière Hospital, Paris);

Sleep assessment: Isabelle Arnulf, MD, PhD (Pitié-Salpêtrière Hospital, Paris, coordination), Smaranda Leu Semenescu, MD (Pitié-Salpêtrière Hospital, Paris), Pauline Dodet, MD (Pitié-Salpêtrière Hospital, Paris);

Genetic data: Jean-Christophe Corvol, MD, PhD (Pitié-Salpêtrière Hospital, Paris, coordination), Graziella Mangone, MD, PhD (Pitié-Salpêtrière Hospital, Paris, co-coordination), Samir Bekadar, MS (Pitié-Salpêtrière Hospital, Paris, biostatistician), Alexis Brice, MD (ICM, Pitié-Salpêtrière Hospital, Paris), Suzanne Lesage, PhD (INSERM, ICM, Paris, genetic analyses);

Metabolomics: Fanny Mochel, MD, PhD (Pitié-Salpêtrière Hospital, Paris, coordination), Farid Ichou, PhD (ICAN, Pitié-Salpêtrière Hospital, Paris), Vincent Perlbarg, PhD, Pierre and Marie Curie University), Benoit Colsch, PhD (CEA, Saclay), Arthur Tenenhaus, PhD (Supelec, Gif-sur-Yvette, data integration);

Brain MRI data : Stéphane Lehericy, MD, PhD (Pitié-Salpêtrière Hospital, Paris, coordination), Rahul Gaurav, MS, (Pitié-Salpêtrière Hospital, Paris, data analysis), Nadya Pyatigorskaya, MD, PhD, (Pitié-Salpêtrière Hospital, Paris, data analysis); Lydia Yahia-Cherif, PhD (ICM, Paris, Biostatistics), Romain Valabregue, PhD (ICM, Paris, data analysis), Cécile Galléa, PhD (ICM, Paris);

Datscan imaging data: Marie-Odile Habert, MCU-PH (Pitié-Salpêtrière Hospital, Paris, coordination);

Voice recording: Dijana Petrovska, PhD (Telecom Sud Paris, Evry, coordination), Laetitia Jeancolas, MS (Telecom Sud Paris, Evry);

Study management: Vanessa Brochard (Pitié-Salpêtrière Hospital, Paris, coordination), Alizé Chalançon (Pitié-Salpêtrière Hospital, Paris, project manager), Carole Dongmo-Kenfack (Pitié-Salpêtrière Hospital, Paris, clinical research assistant);

Study sponsoring: INSERM, Paris
